# Molecular epidemiology of meningococcal disease in England and Wales 1975–1995, before the introduction of serogroup C conjugate vaccines

**DOI:** 10.1099/mic.0.2007/014761-0

**Published:** 2008-04

**Authors:** Joanne E. Russell, Rachel Urwin, Stephen J. Gray, Andrew J. Fox, Ian M. Feavers, Martin C. J. Maiden

**Affiliations:** 1 Peter Medawar Building for Pathogen Research and Department of Zoology, University of Oxford, OX1 3SY, UK; 2 Division of Bacteriology, National Institute for Biological Standards and Control, Blanche Lane, South Mimms, Herts EN6 3QG, UK; 3 Department of Biology, Pennsylvania State University, University Park, PA 16802, USA; 4 Meningococcal Reference Unit, Health Protection Agency, Manchester Royal Infirmary, Oxford Road, Manchester M13 9WZ, UK

## Abstract

A comprehensive meningococcal vaccine is yet to be developed. In the absence of a vaccine that immunizes against the serogroup B capsular polysaccharide, this can only be achieved by targeting subcapsular antigens, and a number of outer-membrane proteins (OMPs) are under consideration as candidates. A major obstacle to the development of such a vaccine is the antigenic diversity of these OMPs, and obtaining population data that accurately identify and catalogue these variants is an important component of vaccine design. The recently proposed meningococcal molecular strain-typing scheme indexes the diversity of two OMPs, PorA and FetA, that are vaccine candidates, as well as the capsule and multilocus sequence type. This scheme was employed to survey 323 meningococci isolated from invasive disease in England and Wales from 1975 to 1995, before the introduction of meningococcal conjugated serogroup C polysaccharide vaccines in 1999. The eight-locus typing scheme provided high typeability (99.4 %) and discrimination (Simpson's diversity index 0.94–0.99). The data showed cycling of meningococcal genotypes and antigenic types in the absence of planned interventions. Notwithstanding high genetic and antigenic diversity, most of the isolates belonged to one of seven clonal complexes, with 11 predominant strain types. Combinations of PorA and FetA, chosen on the basis of their prevalence over time, generated vaccine recipes that included protein variants found in 80 % or more of the disease isolates for this time period. If adequate immune responses can be generated, these results suggest that control of meningococcal disease with relatively simple protein component vaccines may be possible.

## INTRODUCTION

The lack of a comprehensive vaccine for protection against meningococcal disease has been highlighted by the development and introduction of conjugate polysaccharide vaccines against serogroups A, C, Y and W-135 (Jodar *et al.*, 2002). Serogroup B, the predominant cause of meningococcal disease in many countries (Caugant, 1998), is not, therefore, covered by routine immunization and the development of a conjugate vaccine against this polysaccharide is hampered by low immunogeniticy and perceived safety issues (Stein *et al.*, 2006). Both these problems are a consequence of the identity of this molecule with host polysaccharides (Finne *et al.*, 1983). Various approaches that use subcapsular antigens, both proteins and polysaccharides, have been proposed to provide what are best described as serogroup B substitute meningococcal vaccines (Jodar *et al.*, 2002).

Any meningococcal vaccine must accommodate the extensive genetic and antigenic diversity of this common inhabitant of the human nasopharynx (Stephens, 2007). Two contrasting approaches have been employed: the identification of conserved antigens; or the generation of specific vaccines against certain types. The former includes the identification of candidate antigens from genome sequence data, so-called reverse vaccinology (Pizza *et al.*, 2000), while the latter is exemplified by the use of outer-membrane vesicle (OMV) vaccines to disrupt outbreaks of serogroup B disease caused by a single meningococcal clone (Bjune *et al.*, 1991; O'Hallahan *et al.*, 2004; Sierra *et al.*, 1991). In practice the identification of highly conserved antigens has proved difficult, probably because any surface component that is expressed at sufficient levels to be a vaccine target is naturally variable. There remains the possibility of either making specific vaccines that target particular meningococcal variants or producing multivalent formulations of antigens that achieve broad coverage (van den Dobbelsteen *et al.*, 2007). Both these approaches require detailed information about the molecular epidemiology of the meningococcus (Perrett & Pollard, 2005).

Despite their high diversity, meningococcal populations are structured into lineages that are identified as clonal complexes (cc) by multilocus sequence typing (MLST) (Maiden *et al.*, 1998). Only a minority of these, the so-called hyperinvasive lineages, are regularly associated with human disease (Caugant, 2001; Maiden *et al.*, 1998; Yazdankhah *et al.*, 2004). The prevalence of these lineages varies geographically and temporally but each complex tends to be associated with a particular repertoire of surface antigens (Urwin *et al.*, 2004). Three strain-specific vaccines, based on OMV preparations, have been developed and deployed in response to particular outbreaks of meningococcal disease (Oster *et al.*, 2005; Perkins *et al.*, 1998). A more general approach is to identify combinations of meningococcal surface protein variants that will protect against all or at least a high proportion of meningococcal disease; however, the success of such vaccines will depend on the stability of association of antigen variants with invasive meningococci over time (Sacchi *et al.*, 2000).

In the present work, molecular typing, based on the nucleotide sequence determination of meningococcal genes (Elias *et al.*, 2006; Jolley *et al.*, 2007), was employed to identify the variants of two major surface proteins, PorA and FetA, in England and Wales over 20 years prior to the introduction of meningococcal serogroup C conjugate (MCC) vaccines (Miller *et al.*, 2001). These proteins are both typing targets and components that have been present in a number of strain-specific meningococcal vaccines (Jodar *et al.*, 2002). In addition, the clonal complex and hence the hyperinvasive lineage was determined by MLST (Maiden *et al.*, 1998). The data produced a highly discriminatory typing scheme but, notwithstanding a very high diversity of strain types, a relatively simple vaccine based on combinations of PorA and FetA variants could potentially protect against a majority of meningococcal disease over this extended period of time.

## METHODS

### Bacterial isolates.

A total of 125 isolates had been stored from 1975, comprising all of the isolates submitted to the Health Protection Agency England (HPA, formerly Public Health Laboratory Service England and Wales, PHLS) central reference laboratory in 1975. Although they did not necessarily provide a representative sample of the disease at that time, this is one of the largest isolate collections, with accompanying documentation, for one country from that time. By 1985 the more systematic submission of isolates to the HPA (at that time PHLS, England and Wales) Meningococcal Reference Unit (MRU) was common and a subset of 100 representative isolates was chosen from these. For 1995, when the MRU received nearly all meningococci isolated from notified cases of invasive disease, a structured sample was generated by choosing every tenth isolate submitted for inclusion in the survey, up to a total of 100 isolates. This gave a total of 325 stored isolates included in the study, which were cultured on heated-blood agar plates overnight. A total of 323 of these isolate were cultivatable: two of the 1975 isolates failed to grow. The overnight growth was used to prepare DNA using an Isoquick extraction kit (Orca Research). Sequence type (ST) information was gained from the two non-viable isolates by extraction of DNA from the stored material.

### MLST.

This was performed as described previously (Jolley *et al.*, 2000). Briefly, the seven housekeeping loci used in the scheme were amplified by PCR and the amplicons purified by precipitation to remove unincorporated amplification primers and nucleotides. These were then used as templates for dideoxynucleotide sequence reactions using BigDye Terminators (ABI). The extension reactions were separated on an automated DNA analyser (ABI) and the sequences assembled with the Staden software package (Staden, 1996).

### Antigen gene sequencing.

The nucleotide sequence determination of the regions of the *porA* gene that encode most of the antigenic variability of this protein, VR1 and VR2, was undertaken using previously published methods (Suker *et al.*, 1994). The region of the FetA gene that encodes the principal variable region (VR) of this protein was similarly determined (Thompson *et al.*, 2003). For both proteins the peptide sequences were deduced and assigned names using the published nomenclature schemes (Jolley *et al.*, 2007; Russell *et al.*, 2004; Thompson *et al.*, 2003).

### Data storage and analysis.

Data were stored on a customized isolate database available at http://pubmlst.org/neisseria/. The database automatically generated the strain types according to the recently proposed typing nomenclature (Jolley *et al.*, 2007). This has the format serogroup : PorA type : FetA type : sequence type (clonal complex); for example B : P1.7-2,4 : F1-5 : ST-41 (cc41/44). Analysis was conducted with the PubMLST database tools (Jolley & Maiden, 2006; Jolley *et al.*, 2004). The diversity index for strain types was calculated according to Simpson (1949) and modified for strain typing (Hunter & Gaston, 1988).

## RESULTS

### Diversity of strain types

Serogroup information was available for 313 (96 %) of the meningococci examined and complete molecular characterization for MLST, *porA* and *fetA* loci for 323 (99 %) isolates. The two incompletely characterized isolates were the non-viable isolates from the 1975 sample; these were excluded from further analysis. A total of 204 unique strain types were present among the 323 isolates, providing diversity indices of 0.94 for the 1975 isolates and 0.99 for the 1985 and 1995 isolate collections. With the exception of serogroup, of which only four were present in each collection, the isolate collection from 1975 was less diverse than those for 1985 and 1995 for each of the characteristics measured (Table 1). The complete dataset, including geographical distribution of isolates, is available at http://pubmlst.org/neisseria.

### Prevalence of serogroups and clonal complexes over time

Most isolates were serogroup B (71 % of isolates in 1975, 67 % of isolates in 1985, and 58 % of isolates in 1995), with serogroup C the next most prevalent, rising from 20 % of isolates in 1975 through 27 % of isolates in 1985, to 36 % of isolates in 1995. Serogroup A represented 10 % of the isolates in 1975 and 2 % in 1985: it was not present in the 1995 sample. Most serogroup W-135 isolates were present in 1975 (6.5 % of isolates) and most serogroup Y in 1985 (4 % of isolates).

Each serogroup was mainly associated with particular clonal complexes in a given year, but this changed over time (Table 2). There were differences in the clonal complexes present in each of the three years, and the disappearance of serogroup A disease from the UK over the duration of the survey was due to the disappearance of members of the ST-1 complex. Serogroup A is mostly found in members of the ST-1, ST-4 and ST-5 complexes, which rarely express capsules of other serogroups, and their disappearance from most industrialized countries over the period of the survey is yet to be satisfactorily explained (Achtman, 1997). In contrast, the serogroups expressed by other clonal complexes changed during this period. For example, the percentage of isolates that were serogroup C increased from 20 % to 36 % but whereas most of these isolates belonged to ST-344 complex in 1975, the ST-11 complex was the major cause of serogroup C disease in 1995; members in this clonal complex predominantly expressed serogroup W-135 in 1975. In 1975 most serogroup B meningococcal disease was caused by ST-8 isolates; this was succeeded by members of the ST-32 complex in 1985 and members of the ST-41/44 complex in 1995 (Table 2).

### Prevalence of strain types over time

A total of 12 (5 %) of the strain types were observed five times or more in the isolate collection, accounting for 93 (30 %) of all isolates. Of the remaining strain types, 168 (82 %) were observed only once (48/125 in 1975, 51/100 in 1985, and 68/100 in 1995) and 15 (7 %) types were observed twice: two in 1975 and 1985 [B : P1.7,16-2 : F1-5 : ST-32 (cc32) and B : P1.7-2,13-1 : F3-9 : ST-8 (cc8)] and two in 1985 and 1995 [B : P1.19,15 : F5-1 : ST-34 (cc32) and C : P1.5,2 : F5-8 : ST-11 (cc11)]. Of the eight strain types observed three times (4 %), only one occurred in two years [B : P1.19-1,15-11 : F1-7 : ST-269 (cc269) in 1985 and 1995)] and both (1 %) of the types observed four times (2 %) were limited to one year. Together with isolates belonging to the same clonal complex, the most prevalent strain types accounted for between 76 % and 88 % of the disease isolates in any one year, although the prevalence of strain types varied with time (Table 3).

### Distribution of antigen variants

**Fig. 1. f1:**
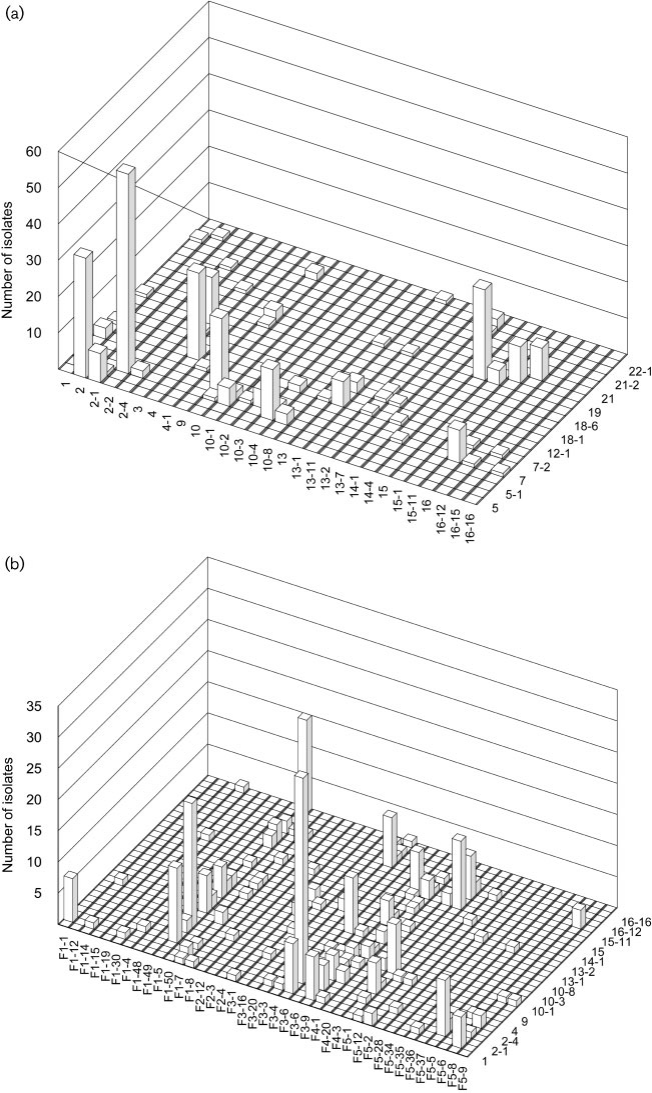
Prevalence of PorA and FetA VR variants from 1975 to 1995. (a) The number of isolates with particular PorA VR1 and VR2 combinations. (b) The number of isolates with particular PorA VR2 and FetA VR combinations.

**Fig. 2. f2:**
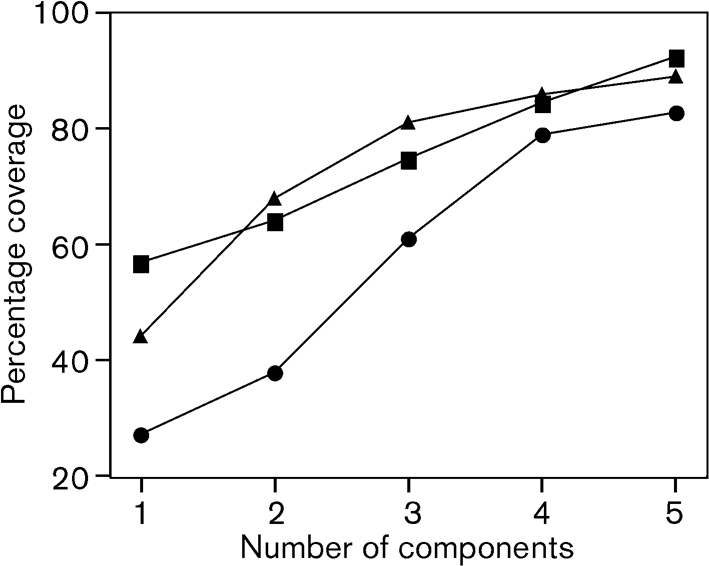
Potential coverage of a PorA/FetA combination vaccines in 1975 (▪), 1985 (•) and 1985 (▴).

There were 131 unique combinations of PorA VR1, PorA VR2 and FetA VR, including three isolates that could not be characterized for PorA. Of the remaining 128 combinations, 85 occurred only once. Of the 43 combinations observed more than once, 30 were only seen in one year, ten in two years and three in all three years. The two most frequent combinations in the whole dataset were each observed in only one year: P1.5-1,2-2 : F3-6, which was present in 27 % of the 1975 isolates, and P1.7-2,4 : F1-5, which was present in 8.9 % of the 1995 isolates. The most prevalent PorA VR1 variant was 7-2, the most prevalent PorA VR2 variant was 2-2 and the most prevalent FetA VR was F1-5 (Table 4). The combined prevalence of the five most common variants was 68 % for PorA VR1, 48 % for PorA VR2, and 66 % for FetA VR. There were a limited number of combinations of the different VRs in the dataset with a tendency for non-overlapping structure (Gupta *et al.*, 1996), although this was more marked with the two PorA VRs than with the PorA VRs and FetA VR (Fig. 1). The data permitted the design of a possible combination of PorA and FetA VRs for use in a vaccine, and the assessment of possible levels of coverage that such a vaccine might be able to attain, in the absence of immunological constraints (Fig. 2).

## DISCUSSION

Public health interventions against variable pathogenic bacteria require accurate, portable and reproducible typing schemes. This is especially important if, as with the meningococcus, the bacterium is not an obligate pathogen because the identification of invasive types can affect the nature of the intervention deployed (Maiden, 2002, 2004). MLST has provided a very reliable means of identifying hyperinvasive meningococci (Brehony *et al.*, 2007), but does not necessarily provide sufficient discrimination for the unambiguous identification of disease clusters representing outbreaks (Bygraves *et al.*, 1999; Feavers *et al.*, 1999). The addition of PorA and FetA VRs added discrimination and identified antigen variants in this sample of invasive meningococci (Jolley *et al.*, 2007) and such ‘fine typing’ has been employed in the identification of outbreak clusters in Germany (Elias *et al.*, 2006).

The levels of discrimination achieved in the current survey were high (Simpson's diversity index 0.94–0.99), comparable with the result achieved with PorA and FetA typing previously, 0.96 (Elias *et al.*, 2006). They were also similar to the best levels of coverage and precision achieved with other methods, indicating that the nine-gene sequence typing scheme has sufficient discrimination for the reliable detection of outbreaks (Swaminathan *et al.*, 1996). In addition, the clonal complex information identified hyperinvasive lineages, enabling the monitoring of trends in meningococcal disease over time and comparison with other datasets (Brehony *et al.*, 2007). Furthermore, as the procedure is PCR based it is inherently suitable for application to clinical specimens (Taha *et al.*, 2005), and finally, the typing data were also informative as to the antigenic variability of the meningococcus.

The lower estimate of diversity index from the 1975 dataset was probably due to the limitations in geographical sampling of disease isolates at this time. Most of the isolates present in the collections for the two later years were independent. However, as the 1975 sample was the complete set of isolates available from submissions to the PHLS, it is possible that epidemiologically related isolates were present, which could also explain the high proportion of ST-8 isolates; indeed it is perhaps more likely that outbreak isolates would have been submitted to the PHLS reference facilities at this time. Geographical source of isolation was available for all of the 1975 and 1995 isolates and for 93 of the 1985 isolates. Of the 48 locations that submitted isolates in 1975, 12 submitted four or more with the greatest (Manchester) submitting 20. By contrast, in the 1985 dataset 55 locations were represented, only two of which (London, eight and Manchester, four) were represented more than three times. Similarly, in the 1995 dataset of 67 locations, only London and Manchester (five isolates each) were represented by more than three isolates.

The data were consistent with previous studies that have indicated dynamic behaviour in meningococcal populations (Caugant, 2001; Harrison *et al.*, 2006; Yazdankhah *et al.*, 2004). Disease incidence rises and falls with the presence of hyperinvasive lineages in the carried population of meningococci. The antigens associated with particular clonal complexes consequently rise and fall over time, as predicted by models of pathogen strain structuring by immunological selection (Gupta & Maiden, 2001), although there appeared to be differences in the stability of the lineages with regard to different antigens. The ST-1 clonal complex is very strongly associated with particular antigenic variants, including capsule and subcapsular antigens (Suker *et al.*, 1994; Urwin *et al.*, 2004), and serogroup A disease disappeared from the UK with this clonal complex. Despite reintroduction since then, for example with ST-5 complex meningococci in the 1990s, serogroup A meningococci have not, to date, re-established themselves as a cause of disease in the UK (Jones & Sutcliffe, 1990). The sialic-acid-based capsules, corresponding to serogroups B, C, Y and W-135, have a more dynamic relationship with clonal complex. This may reflect the fact that horizontal gene exchange of only the gene occupying the *siaD* locus of the capsular region is required to alter the serogroup (Swartley *et al.*, 1997; Vogel *et al.*, 2000). A further difference is that the serogroup A capsule is thought to be more immunogenic (Gold *et al.*, 1979).

The clonal complexes associated with capsules containing sialic acid (the ST-8, ST-32, ST-344 and ST-41/44 complexes) are, however, more limited in the repertoire of subcapsular antigens that they express, each being associated with particular variant families. In particular, there was a tendency of the VRs to occur in non-overlapping combinations (Fig. 1), which, along with the dynamic behaviour noted above, is consistent with models of strain structuring based on herd immunity (Gupta & Maiden, 2001; Gupta *et al.*, 1996). This also confirmed the observations made on a global dataset, where it was suggested that combinations of PorA VRs and FetA VRs might be an effective means of controlling meningococcal disease (Urwin *et al.*, 2004). Simple vaccines based on combinations of these two components could achieve high coverage of circulating meningococci (Fig. 2). The implementation of such a vaccine may require epidemiological monitoring to ensure that it remains effective (Trotter *et al.*, 2007).

Current approaches in the development of a comprehensive meningococcal vaccine can be divided into two distinct strategies: those seeking to enhance the immune response to conserved antigens and those based on highly immunogenic yet variable antigens. While not excluding the utility of vaccines based on conserved antigens, the observations reported here support the development of multivalent formulations consisting of variable antigens. The evidence of strain structuring indicates that such antigens are the principal targets of natural immunity. This is also consistent with clinical studies of OMV vaccines, which have repeatedly highlighted the importance of PorA for vaccine-induced immunity. In terms of clinical development, the most advanced multivalent vaccines consist of OMVs produced from genetically modified strains expressing multiple variants of PorA (van den Dobbelsteen *et al.*, 2007). The present study provides a rational approach for deciding which variants should be included in this type of vaccine. It further suggests that ensuring the expression of appropriate variants of a second antigen, such as FetA, would be an effective way of broadening vaccine coverage.

In conclusion, meningococci isolated from cases of invasive disease are highly diverse at both housekeeping protein and antigen encoding loci; however, much of this diversity is transient, especially the minor variants of protein antigens. A more limited repertoire of antigen variants is persistent over time and these tend to be associated with particular invasive lineages. Combinations of subcapsular antigens reappear over time, sometimes associated with different lineages, perhaps in response to increases and decreases of herd immunity against particular strain types. This leads to the possibility that appropriately composed component vaccines may be able to protect human populations from meningococcal disease over periods of time sufficient to warrant their development and implementation.

## Figures and Tables

**Table 1. t1:** Diversity of meningococci isolated from invasive disease over 20 years in England and Wales

	**Number**		
	**1975**	**1985**	**1995**
Isolates	125	100	100
Sequence typed	123	100	100
Strain types	67	70	79
Serogroups	4	4	4
PorA VR1 variants	15	18	13*
PorA VR2 variants	20	25	21
FetA VR variants	23	19	22
STs	48	55	52
Clonal complexes	13	18	11
Isolates not assigned to a complex	4	5	6

*Including one isolate with a stop codon in VR1.

**Table 2. t2:** Association of serogroups with predominant clonal complexes over time

**Clonal complex**	**Prevalence (%)**											
	**1975**				**1985**				**1995**			
	**A**	**B**	**C**	**W-135**	**A**	**B**	**C**	**W-135**	**A**	**B**	**C**	**W-135**
ST-1 complex	9.2				2							
ST-8 complex		38				4	6			2	6	
ST-11 complex		0.8		4.9			7			4	27	
ST-32 complex		0.8				31				7		
ST-41/44 complex		5.7				11	1			25	1	
ST-334 complex		2.4	16			3	4					
ST-269 complex		0.8				7				12		

**Table 3. t3:** Prevalence of strain types represented more than five times and strain types belonging to the same clonal complex

**Strain type**	**Prevalence (%)**			
	**1975**	**1985**	**1995**	**Mean**
**ST-8 complex**				
B : P1.5-1,2-2 : F3-6 : ST-8 (cc8)	24			8.1
B : P1.19,15 : F3-9 : ST-8 (cc8)	3.3	1		1.4
23 other cc8 strain types	12	9	8	9.7
**ST-41/44 complex**				
B : P1.7-2,4 : F1-5 : ST-41 (cc41/44)		3	6	3
40 other cc41/44 strain types	6.5	9	22	10
**ST-11 complex**				
C : P1.5,2-1 : F5-5 : ST-11 (cc11)		6	2	2.7
C : P1.5,2 : F3-6 : ST-11 (cc11)			6	2
C : P1.5-1,10-4 : F3-6 : ST-11 (cc11)			6	2
21 other cc11 strain types	5.7	1	18	8.7
**ST-32 complex**				
B : P1.19,15 : F5-1 : ST-33 (cc32)		6	1	2.3
B : P1.7,16-2 : F1-5 : ST-74 (cc32)		4	2	2
B : P1.7,16-2 : F1-5 : ST-343 (cc32)		5		1.7
15 other cc32 strain types	0.8	16	5	7.3
**ST-1 complex**				
A : P1.5-2,10 : F5-1 : ST-1 (cc1)	4	1		2
5 other cc1 strain types	5.7	1		2.7
**ST-334 complex**				
C : P1.5-1,2-2 : F1-5 : ST-334 (cc334)	3.3	1		1.7
23 other cc334 strain types	15.5	6		8.3
**ST-269 complex**				
B : P1.19-1,15-11 : F5-1 : ST-269 (cc269)		3		1
13 other cc269 isolates	0.8	4	12	5.6
**Total**	82.1	76	88	82

**Table 4. t4:** Most prevalent protein antigen variants

**Rank order**	**Protein variant (prevalence in all three years, %)**		
	**PorA VR1**	**PorA VR2**	**FetA VR**
1	5-1 (23.4)	2-2 (15.2)	F1-5 (23.8)
2	5 (13.1)	2 (11.1)	F3-6 (15.6)
3	7-2 (12.4)	4 (8)	F5-1 (11.7)
4	7 (9.6)	15 (7.6)	F3-9 (9.8)
5	19 (9.6)	10 (6.4)	F4-1 (4.8)
Combined prevalence	68.1	48.3	65.7

## Abbreviations

- MLST, multilocus sequence typing
- OMV, outer-membrane vesicle
- PHLS, Public Health Laboratory Service
- ST, sequence type
- VR, variable region
